# Persistent low‐level viraemia is associated with non‐infectious comorbidities in an observational cohort in four African countries

**DOI:** 10.1002/jia2.26316

**Published:** 2024-08-27

**Authors:** Allahna L. Esber, Suze Colt, Ningbo Jian, Nicole Dear, Bonnie Slike, Valentine Sing'oei, Jonah Maswai, Michael Iroezindu, Emmanuel Bahemana, Hannah Kibuuka, Christina S. Polyak, Hendrik Streeck, Neha Shah, Trevor A. Crowell, Julie A. Ake, the AFRICOS Study Group

**Affiliations:** ^1^ U.S. Military HIV Research Program Walter Reed Army Institute of Research Silver Spring Maryland USA; ^2^ Henry M. Jackson Foundation for the Advancement of Military Medicine Bethesda Maryland USA; ^3^ U.S. Army Medical Research Directorate ‐ Africa Kisumu Kenya; ^4^ HJF Medical Research International Kisumu Kenya; ^5^ U.S. Army Medical Research Directorate ‐ Africa Kericho Kenya; ^6^ HJF Medical Research International Abuja Nigeria; ^7^ HJF Medical Research International Mbeya Tanzania; ^8^ Makerere University Walter Reed Project Kampala Uganda; ^9^ Institute of Virology University Hospital Bonn Germany; ^10^ Institute of HIV Research University Duisburg‐Essen Essen Germany

**Keywords:** cohort studies, HIV, low‐level viraemia, multimorbidity, non‐communicable diseases, sub‐Saharan Africa

## Abstract

**Introduction:**

People living with HIV (PLWH) have higher rates of non‐infectious comorbid diseases (NCDs) than individuals without HIV. We characterized the risk of NCDs among PLWH with undetectable viral load and persistent low‐level viraemia (pLLV) in the African Cohort Study (AFRICOS). We secondarily quantified the role of immune activation in the association between LLV and NCDs.

**Methods:**

AFRICOS enrols participants in 12 clinics in Uganda, Kenya, Tanzania and Nigeria. Participants on antiretroviral therapy ≥ 6 months without an NCD at enrolment were included. PLLV was defined as at least two consecutive visits with a detectable viral load <1000 copies/ml. We examined elevated blood pressure, hypercholesterolemia, hyperglycaemia, renal insufficiency and a composite variable of any NCD. Hazard ratios (HRs) and 95% confidence intervals (CIs) were estimated using Cox proportional hazard modelling. Among a subset of participants with biomarker data, we assessed the interaction between viral load and 13 biomarkers in the association with any NCD.

**Results:**

From 23 January 2013 to 1 December 2022, 1755 participants met the inclusion criteria for these analyses. At the first eligible visit, the majority of participants had an undetectable viral load (*n* = 1375, 78.35%). Participants with pLLV had an increased rate of developing any NCD (aHR: 1.22, 95% CI: 1.02−1.47) compared to participants with an undetectable viral load. There was a statistically significant interaction between LLV and TNF‐α, CCL2/MCP‐1 and TNF‐RII in the association with any NCD.

**Conclusions:**

PLLV was significantly associated with NCDs and immune inflammation in this population. Aggressive management of LLV may positively impact NCDs in PLWH.

## 1 INTRODUCTION

Despite improved life expectancy with antiretroviral therapy (ART), people living with HIV (PLWH) have higher rates of non‐infectious comorbid diseases (NCDs) than do people living without HIV [1, 2, 3, 4, 5]. The interplay between HIV and NCDs is not yet completely characterized. PLWH experience a greater burden of traditional risk factors for NCDs such as smoking and alcohol use [1]. Additionally, some ARTs have been associated with excess weight gain, elevated blood pressure (BP) and renal insufficiency [3, 6, 7]. Previous research has also shown an association between persistent low‐level viraemia (pLLV) and certain inflammatory markers and a potential link between pLLV and cardiovascular disease [8].

Previously, pLLV as low as 50 copies/ml has been associated with viral failure, serious non‐AIDS events and mortality [9, 10, 11]. Chronic inflammation and immune activation due to persistent low‐level viral replication may contribute to the heightened risk of NCDs among some PLWH. Ongoing viral replication can cause inflammation that is associated with cardiovascular disease, metabolic disorders, neurocognitive decline, bone complications and frailty [5, 12, 13, 14].

Previous research has cross‐sectionally explored the associations between markers for immune activation and hypercholesterolemia, hypertension, hyperglycaemia and renal injury. Findings were mixed with some markers, such as CD25, having a protective association with NCDs while others, including IL‐6, CXCL10 and CD163, were associated with an increased odds of NCDs [15, 16, 17]. In one study examining the same population as this analysis, they found that among participants with viral load ≥50 c/ml all immune parameters significantly correlated with viral load with the exception of the antiviral cytokine, IFN‐α [15].

Our primary objective was to longitudinally assess the risk of developing several NCDs, including elevated BP, hypercholesterolemia, hyperglycaemia and renal insufficiency, among PLWH with undetectable plasma viral load and pLLV in the African Cohort Study (AFRICOS). We secondarily quantified the interaction of low‐level viraemia (LLV) and immune activation markers on NCDs among a subset of AFRICOS participants enrolled between January 2013 and December 2017 with available biomarker data.

## 2 METHODS

### 2.1 Cohort description

The African Cohort Study enrols PLWH at 12 PEPFAR‐supported clinical sites in Uganda, Kenya, Tanzania and Nigeria. Enrolment began in January 2013 and is ongoing. Participants provide a medical history, complete a physical examination and undergo laboratory assessments at enrolment and every 6 months. Participants are recruited from current clinic clients and newly diagnosed individuals. A small subset (9%) is recruited from previous clinical trials located at the site. All non‐pregnant, current clinic clients, 18 years and older, and consenting to data and specimen collection are eligible for inclusion in AFRICOS. Beginning in January 2020, eligibility expanded to individuals 15–17 years of age.

### 2.2 Primary exposure

The main exposure was pLLV defined as at least two consecutive visits with a detectable viral load <1000 copies/ml. Viral load was quantified at enrolment and each 6 monthly visit using standard polymerase chain reaction‐based clinical tests at each site [18]. The lower limit of detection ranged from 20 to 48 copies/ml dependent on the time of measurement and study site. For example, if a participant had a viral load of 50 c/ml at the first study visit followed by a viral load of 750 c/ml at the second study visit, they would be classified as having pLLV for those study visits. If at the third visit, the participant had an undetectable viral load, they would then be classified as undetectable for the third study visit.

### 2.3 Outcome

We examined four different NCDs—elevated BP, hypercholesterolemia, hyperglycaemia and renal insufficiency. Elevated BP was defined as any single systolic pressure >139, diastolic >89 mmHG or use of anti‐hypertensive medications. For participants with elevated measurements, repeat results were taken at the same visit. Hypercholesterolemia was defined as a total cholesterol >199 mg/dl or the use of lipid‐lowering medications. Hyperglycaemia was defined as any non‐fasting glucose >199 mg/dl, fasting glucose >99 mg/dl or use of diabetes medication, and renal insufficiency was defined as an estimated glomerular filtration rate <60 ml/minute/1.73 m^2^ calculated using the Modification of Diet in Renal Disease equation based on serum creatinine. We also included a composite NCD variable assessing whether participants had any or none of the four NCDs.

### 2.4 Primary analysis

In the main analysis, examining the rate of developing NCDs by viral load category, we included participants on ART for at least 6 months who had available viral load and NCD data. Participants with an NCD at the enrolment visit were excluded. Visits with a viral load >1000 c/ml were excluded but if participants had other study visits with a viral load <1000 c/ml, those visits were included. The viral load category was time‐varying and thus participants could contribute time to multiple categories over the course of follow‐up. Cox proportional hazards modelling was used to estimate hazard ratios (HRs) and 95% confidence intervals (95% CIs). Age, study site, body mass index (BMI), cigarette use, ART adherence, ART regimen (efavirenz, nevirapine, dolutegravir, protease inhibitor [PI], other) and education were included as confounders in the adjusted models. Given the strong association with ART and both the exposure and outcome, we ran a sensitivity analysis repeating the analysis restricting to only participants on dolutegravir. To assess whether participants who went on to viral failure after pLLV were influencing results, we ran an additional sensitivity analysis excluding these participants. The assumption of proportional hazards was assessed using Schoenfeld residuals.

### 2.5 Secondary exposure

For the secondary analysis, we included 13 different biomarkers in the standard Luminex Screening Assay‐IL‐6, CXCL10, IL‐10, CCL2, IL‐1β, IFN‐γ, MIP‐1β, CD163, CD25, CXCL9, TNF‐α, TNF RII and IFN‐α. Cytokine/chemokine profiling by Luminex Biomarkers was measured using a Luminex Screening Assay (R&D Systems, Minneapolis, MN) according to the manufacturer's protocol. IFN‐α measurement was performed using VeriKine^TM^ Human IFN Alpha ELISA kit®(PBL Assay Science, Piscataway, NJ), following the manufacturer's instructions.

### 2.6 Secondary analysis

As a secondary analysis, we examined the interaction between LLV and immune activation markers in the association with any NCD, elevated BP, hypercholesterolemia, hyperglycaemia and renal insufficiency at the enrolment visit. Biomarker data were only available at the enrolment visit for participants enrolled between January 2013 and December 2017. Among that subset, only participants who were on ART for at least 6 months with a viral load <1000 copies/ml were included and categorized as either undetectable or LLV (any detectable viral load<1000 copies/ml). Descriptively, we explored significant differences in median biomarker concentration by viral load category using the Wilcoxon ranked test. Bonferroni corrections were made to adjust for multiple comparisons. We created an interaction term between viral load category and each of the 13 biomarkers for any NCD and separately for hypercholesterolemia, hyperglycaemia, elevated BP and renal insufficiency using modified Poisson regression with robust standard errors.

The study was approved by the institutional review boards of the Walter Reed Army Institute of Research, Makerere University School of Public Health, Kenya Medical Research Institute, Tanzania National Institute of Medical Research and the Nigerian Ministry of Defence. All participants provided written informed consent.

## 3 RESULTS

From January 2013 to 1 December 2022, 3348 participants were enrolled into AFRICOS, with 2334 participants not having any NCDs at enrolment. Of those, 1755 participants had study visits with at least 6 months on ART and without viral failure and were included in this analysis. Participants contributed a median follow‐up time of 2.01 years (interquartile range [IQR]: 0.99−4.65 years) and five viral load results (IQR: 3−10). At the first eligible visit, the majority of participants had an undetectable viral load (*n* = 1375, 78.35%) and were female (*n* = 1004, 57.21%; Table 1). Viral load differed significantly by ART regimen with a higher percentage of participants on dolutegravir with viral suppression (*n* = 475, 87.48%) as compared to efavirenz (*n* = 599, 73.41%), nevirapine (*n* = 203, 80.88%) and PIs (*n* = 80, 65.04%; *p*<0.001). Across all included visits, 28.42% were pLLV.

**Table 1:** Select participant characteristics at the first eligible study visit by viral load category

	All participants	Undetectable	Persistent low‐level viraemia	*p*‐value
	(*n*=1755)	(*n*=1375)	(*n*=380)	
**Study site**				<0.001
Uganda	393 (22.4%)	252 (18.3%)	141 (37.1%)	
SRV, Kenya	576 (32.8%)	518 (37.7%)	58 (15.3%)	
Kisumu West, Kenya	357 (20.3%)	315 (22.9%)	42 (11.1%)	
Tanzania	284 (16.2%)	188 (13.7%)	96 (25.3%)	
Nigeria	145 (8.3%)	102 (7.4%)	43 (11.3%)	
**Age (years)**				0.34
<30	549 (31.3%)	436 (31.7%)	113 (29.7%)	
30−39	513 (29.2%)	390 (28.4%)	123 (32.4%)	
40−49	451 (25.7%)	350 (25.5%)	101 (26.6%)	
50+	241 (13.7%)	198 (14.4%)	43 (11.3%)	
**Sex**				0.012
Male	751 (42.8%)	567 (41.2%)	184 (48.4%)	
Female	1004 (57.2%)	808 (58.8%)	196 (51.6%)	
**Education**				0.080
Primary or less	979 (55.8%)	752 (54.7%)	227 (59.7%)	
Secondary or above	776 (44.2%)	623 (45.3%)	153 (40.3%)	
**BMI**				0.29
<18.5	196 (11.2%)	157 (11.4%)	39 (10.3%)	
18.5−24.99	1146 (65.3%)	885 (64.4%)	261 (68.7%)	
25+	413 (23.5%)	333 (24.2%)	80 (21.1%)	
**Ever smoke cigarettes**				0.29
No	1712 (97.5%)	1345 (97.8%)	367 (96.6%)	
Yes	42 (2.4%)	29 (2.1%)	13 (3.4%)	
**ART class**				<0.001
Efavirenz	816 (46.5%)	599 (43.6%)	217 (57.1%)	
Nevirapine	251 (14.3%)	203 (14.8%)	48 (12.6%)	
Dolutegravir	543 (30.9%)	475 (34.5%)	68 (17.9%)	
Protease inhibitor	123 (7.0%)	80 (5.8%)	43 (11.3%)	
Other	22 (1.3%)	18 (1.3%)	4 (1.1%)	
**Missed ART drugs in previous 30 days**				<0.001
None	1473 (83.9%)	1182 (86.0%)	291 (76.6%)	
Any	277 (15.8%)	188 (13.7%)	89 (23.4%)	
Missing	5 (0.3%)	5 (0.4%)	0 (0.0%)	
**Duration on ART**	4.3 (1.6−7.9)	5.0 (1.9−8.3)	2.1 (1.3−5.5)	<0.001

Abbreviations: ART, antiretroviral therapy; BMI, body mass index; SRV, South Rift Valley.

During 5183 person‐years of follow‐up, 899 participants developed one or more NCDs, 488 (28.82%) developed elevated BP, 459 (29.82%) developed hypercholesterolemia, 329 (21.26%) developed hyperglycaemia and 105 (6.35%) developed renal insufficiency. Incidence of any NCD was highest for participants with pLLV with an incidence rate of 287.45 NCDs/1000 person‐years for pLLV (95% CI: 247.22−334.22) compared to 190.15 NCDs/1000 person‐years for undetectable participants (95% CI: 175.01−206.60; Table 2). Among participants with pLLV, 49.83% were undetectable at the subsequent study visit, 8.38% developed viral failure and 41.79% remained with pLLV until the end of follow‐up.

**Table 2:** Incidence rates of select non‐infectious comorbidities by viral load category (per 1000 person‐years)

	Undetectable		pLLV 50−999 copies/ml	
	Incidence	95% CI	Incidence	95% CI
**Any non‐infectious comorbidity**	190.15	175.01−206.60	287.45	247.22−334.22
**Elevated blood pressure**	83.34	74.44−93.31	122.36	98.92−151.34
**Hypercholesterolemia**	79.42	70.55−89.40	131.60	106.66−162.37
**Hyperglycaemia**	51.27	44.47−9.10	92.64	72.51−118.36
**Renal insufficiency**	12.82	9.82−16.74	17.45	10.13−30.06

Abbreviation: CI, confidence interval.

In unadjusted analyses, participants with pLLV had a statistically significant increased risk of developing any NCD (HR: 1.34, 95% CI: 1.12−1.60), hypercholesterolemia (HR: 1.37, 95% CI: 1.07−1.76) and hyperglycaemia (HR: 1.60, 95% CI: 1.19−2.16). After adjustment for confounders, pLLV only remained significantly associated with an increased rate of developing any NCD (aHR: 1.22, 95% CI: 1.02−1.47; Table 3). After excluding the 8.38% of participants who developed viral failure after pLLV findings remained similar (HR for any NCD: 1.46; 95% CI: 1.22−74; aHR: 1.34, 95% CI: 1.11−1.61).

**Table 3:** Adjusted hazard ratios (HR) and 95% confidence intervals comparing persistent low‐level viraemia to virally suppressed participants for select non‐infectious comorbidities

	Any NCD (*n* = 1755)		Elevated blood pressure (*n* = 1693)		Hypercholesterolemia (*n* = 1539)		Hyperglycaemia (*n* = 1547)		Renal insufficiency (*n* = 1651)	
	aHR	95% CI	aHR	95% CI	aHR	95% CI	aHR	95% CI	aHR	95% CI
**Viral load category**										
Undetectable	Ref		−		−		−		−	
pLLV	1.22	1.02−1.47	1.03	0.79−1.34	1.08	0.83−1.40	1.17	0.86−1.60	1.00	0.51−1.93
**Age at visit**										
<30	Ref		−		−		−		−	
30−39	0.98	0.77−1.26	1.12	0.75−1.68	1.02	0.70−1.47	1.14	0.71−1.82	1.41	0.46−4.29
40−49	1.24	0.96−1.59	1.68	1.14−2.49	1.34	0.93−1.93	1.19	0.74−1.91	2.46	0.84−7.17
50+	1.32	1.00−1.75	1.77	1.17−2.68	1.62	1.08−2.43	1.15	0.68−1.94	3.19	1.04−9.83
**Sex**										
Male	Ref		−		−		−		−	
Female	0.78	0.67−0.92	0.58	0.47−0.72	1.15	0.91−1.45	0.69	0.52−0.90	1.60	0.93−2.78
**Education**										
Primary or less	Ref		−		−		−		−	
Secondary or above	1.05	0.90−1.24	1.17	0.94−1.45	1.19	0.94−1.49	0.78	0.59−1.03	1.62	0.93−2.79
**Missed ART drugs in previous 30 days**										
None	Ref		−		−		−		−	
Any	0.99	0.79−1.24	1.08	0.80−1.46	1.19	0.88−1.62	0.90	0.61−1.31	0.87	0.40−1.87
**ART regimen**										
Efavirenz	Ref		−		−		−		−	
Nevirapine	1.19	0.97−1.47	1.59	1.20−2.09	1.16	0.86−1.56	0.96	0.63−1.48	1.96	0.85−4.55
Dolutegravir	0.13	0.10−0.17	0.12	0.09−0.17	0.08	0.05−0.11	0.13	0.09−0.18	0.32	0.17−0.61
Protease inhibitor	0.74	0.56−0.97	0.53	0.36−0.78	0.70	0.49−1.00	0.47	0.29−0.77	1.36	0.61−3.05
Other	0.48	0.26−0.89	0.39	0.17−0.90	0.28	0.13−0.61	0.40	0.17−0.93	1.04	0.23−4.69
**Cigarette use**										
No	Ref		−		−		−			
Yes	1.02	0.66−1.58	0.95	0.1−1.76	1.14	0.58−2.26	1.16	0.54−2.52		
**BMI**										
<18.5	Ref		−		−		−			
18.5−24.99	1.18	0.88−1.58	1.04	0.70−1.55	1.56	1.03−2.37	1.17	0.72−1.90		
25+	1.39	1.01−1.90	1.43	0.93−2.18	1.64	1.05−2.55	1.37	0.82−2.30		
**Study site**										
Uganda	Ref		−		−		−		−	
SRV, Kenya	1.94	1.5−2.42	2.70	1.92−3.78	1.54	1.13−2.11	2.14	1.47−3.11	2.17	0.96−4.94
Kisumu, Kenya	1.37	1.06−1.77	1.71	1.16−2.54	1.60	1.13−2.25	1.05	0.65−1.71	1.20	0.46−3.41
Tanzania	1.97	1.51−2.57	3.41	2.34−4.96	1.21	0.81−1.82	1.32	0.80−2.17	0.82	0.22−3.13
Nigeria	3.22	2.37−4.36	3.07	1.92−4.92	3.21	2.15−4.79	6.00	3.81−9.45	8.27	3.32−20.63

Abbreviations: ART, antiretroviral therapy; BMI, body mass index; CI, confidence interval; HR, hazard ratio; NCD, non‐infectious comorbidity; pLLV, persistent low‐level viraemia; SRV, South Rift Valley.

Restricting to participants on dolutegravir, we see increased HRs in the unadjusted association between pLLV and each NCD. There was a statistically significant increased risk of developing any NCD (HR: 2.93, 95% CI: 1. 59−5.40), elevated BP (HR: 2.33, 95% CI: 1.14−4.75), hypercholesterolemia (HR: 6.51, 95% CI: 2.92−14.51), hyperglycaemia (HR: 2.86, 95% CI: 1.18−6.92) and renal insufficiency (HR: 6.38, 95% CI: 2.52−16.15; Table 4). After adjusting for age, education, ART adherence, sex, cigarette use, BMI and study site, participants with pLLV had a statistically significant increased risk of developing any NCD (aHR: 2.17, 95% CI: 1.10−4.28) and hypercholesterolemia (aHR: 6.78, 95% CI: 2.50−18.37). Similar to the main analysis, findings remained unchanged when excluding participants with pLLV who developed viral failure (HR: 3.11, 95% CI: 1.69−5.72; aHR: 2.40, 95% CI: 1.22−4.74).

**Table 4:** Adjusted hazard ratios (HR) and 95% confidence interval comparing persistent low‐level viraemia to virally suppressed participants for select non‐infectious comorbidities among participants on dolutegravir

	Any NCD (*n* = 627)		Elevated blood pressure (*n* = 762)		Hypercholesterolemia (*n* = 652)		Hyperglycaemia (*n* = 735)		Renal insufficiency (*n* = 924)	
	aHR	95% CI	aHR	95% CI	aHR	95% CI	aHR	95% CI	aHR	95% CI
**Viral load category**										
Undetectable	Ref		−		−		−		−	
pLLV	2.17	1.10−4.28	1.64	0.73−3.68	6.78	2.50−18.37	2.47	0.93−6.56	5.66	1.86−17.23
**Age**										
<30	Ref		−		−		−		−	
30−39	0.67	0.29−1.52	0.73	0.28−1.89	0.52	0.10−2.54	0.94	0.24−3.61	1.30	0.37−4.52
40−49	1.01	0.45−2.25	0.89	0.35−2.28	1.56	0.40−6.03	1.49	0.41−5.36	1.65	0.45−6.07
50+	1.25	0.55−2.83	1.23	0.48−3.17	2.17	0.55−8.54	0.73	1.18−2.99	6.92	1.84−26.01
**Sex**										
Male	Ref		−		−		−		−	
Female	0.76	0.47−1.24	0.47	0.27−0.80	1.25	0.56−2.82	0.79	0.42−1.49	2.95	1.16−7.48
**Education**										
Primary or less	Ref		−		−		−		−	
Secondary or above	0.88	0.52−1.48	1.23	0.72−2.11	1.03	0.46−2.30	0.83	0.43−1.60	1.42	0.60−3.36
**Missed ART drugs in previous 30 days**										
None	Ref		−		−		−		−	
Any	1.27	0.61−2.64	1.64	0.78−3.48	0.89	0.25−3.21	1.88	0.74−4.76	0.94	0.26−3.44
**Cigarette use**										
No	Ref		−		−					
Yes	1.12	0.33−3.76	0.60	0.13−2.65	2.72	0.60−12.31	0.63	0.08−5.05		
**BMI**										
<18.5	Ref		−		−		−			
18.5−24.99	1.01	0.39−2.62	0.58	0.20−1.70	1.84	0.40−8.43	1.66	0.39−7.08		
25+	1.29	0.47−3.56	1.54	0.51−4.66	1.71	0.32−9.05	1.94	0.43−8.75		
**Study site**										
Uganda	Ref		−		−		−		−	
SRV, Kenya	1.93	1.00−3.72	3.01	1.26−7.16	1.64	0.45−5.92	1.08	0.50−2.32	2.07	0.55−7.76
Kisumu, Kenya	0.79	0.36−1.72	0.74	0.24−2.27	3.24	0.89−11.80	0.67	0.26−1.75	1.15	0.26−5.12
Tanzania	3.25	1.61−6.55	7.89	3.23−19.24	4.33	1.20−15.56	0.88	0.27−2.82	0.75	0.08−6.99
Nigeria	6.24	2.35−16.57	4.25	1.35−13.45	6.61	1.36−32.05	1.80	0.45−7.24	11.77	2.82−49.05

### 3.1 Role of immune activation

**Figure 1:**
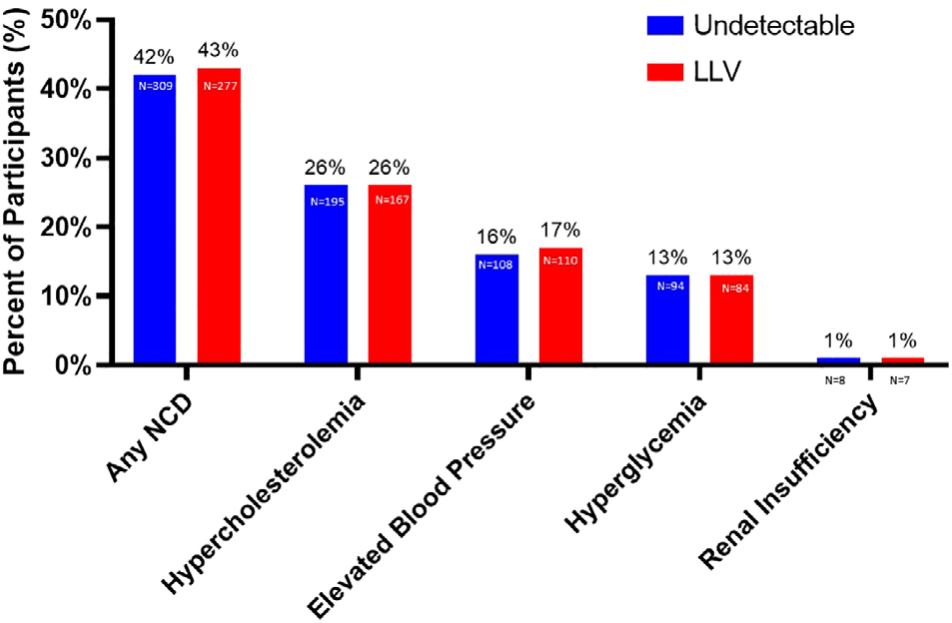
Non‐infectious comorbidities at the enrolment visit by viral load category.

Among the 1384 participants on ART for at least 6 months with available biomarker data, 46.82% (*n* = 648) had LLV at the enrolment visit. At the enrolment visit, 42.34% (*n* = 586) had any NCD, 26.16% (*n* = 362) had hypercholesterolemia, 15.75% had elevated BP (*n* = 218), 12.86% (*n* = 178) hyperglycaemia and 1.08% (*n* = 15) renal insufficiency. There were no statistically significant differences in the prevalence of NCDs by viral load category (Figure 1).

**Figure 2:**
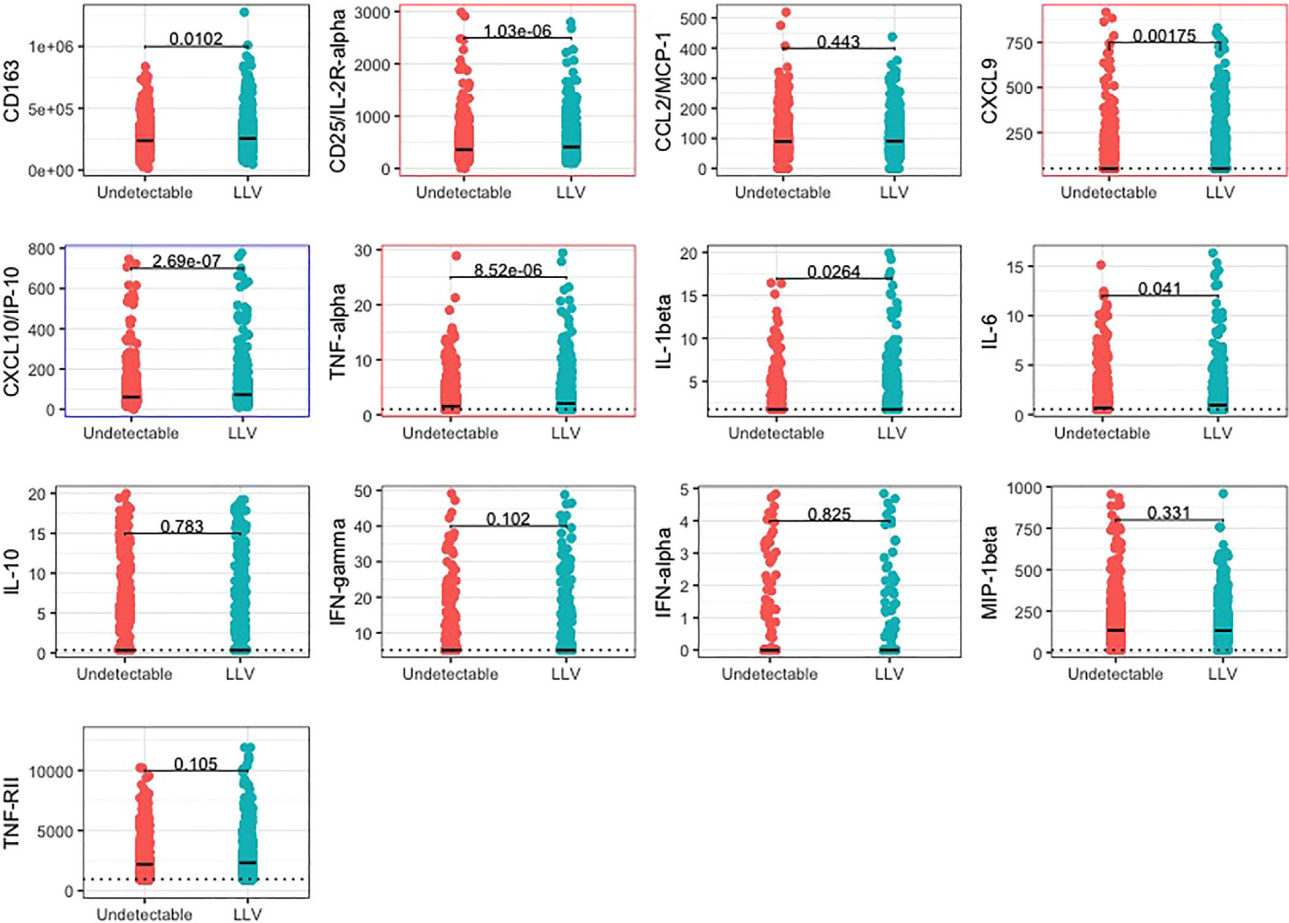
Biomarker concentration by viral load category at the enrolment visit. Dotted line = LLD; black line = median value; red outline = Bonferroni corrected *p*<0.004 positive association between viral load and biomarker concentration; blue outline = Bonferroni corrected *p*<0.004 negative association between viral load and biomarker concentration.

Examining biomarker concentration by viral load category, we saw a statistically significant positive association with higher median concentrations of TNF‐α, CXCL9 and CD25 with LLV compared to those with an undetectable viral load (Figure 2). There was a statistically significant negative association with a lower median concentration of CXCL10‐IP10 with LLV compared to an undetectable viral load.

**Figure 3:**
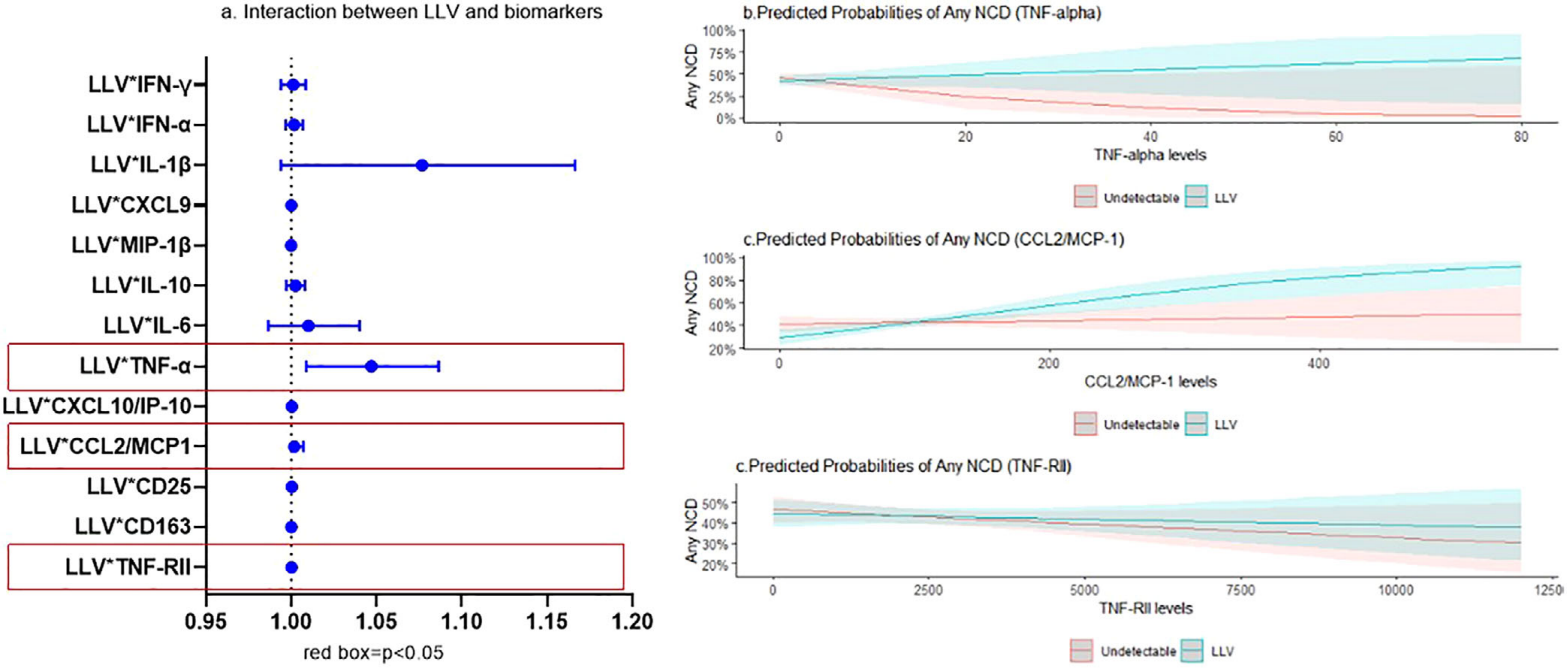
Interaction between low‐level viraemia and immune activation marker in the association with any non‐infectious comorbidity (NCD) at the enrolment visit. (A) Interaction between low‐level viraemia (LLV) and each immune activation marker in the association with any NCD. Each row is a separate model. (B−D) Predicted probability of any NCD by TNF‐α, CCL2/MCP‐1 and TNF‐RII levels comparing participants with undetectable or LLV at the enrolment visit.

**Figure 4:**
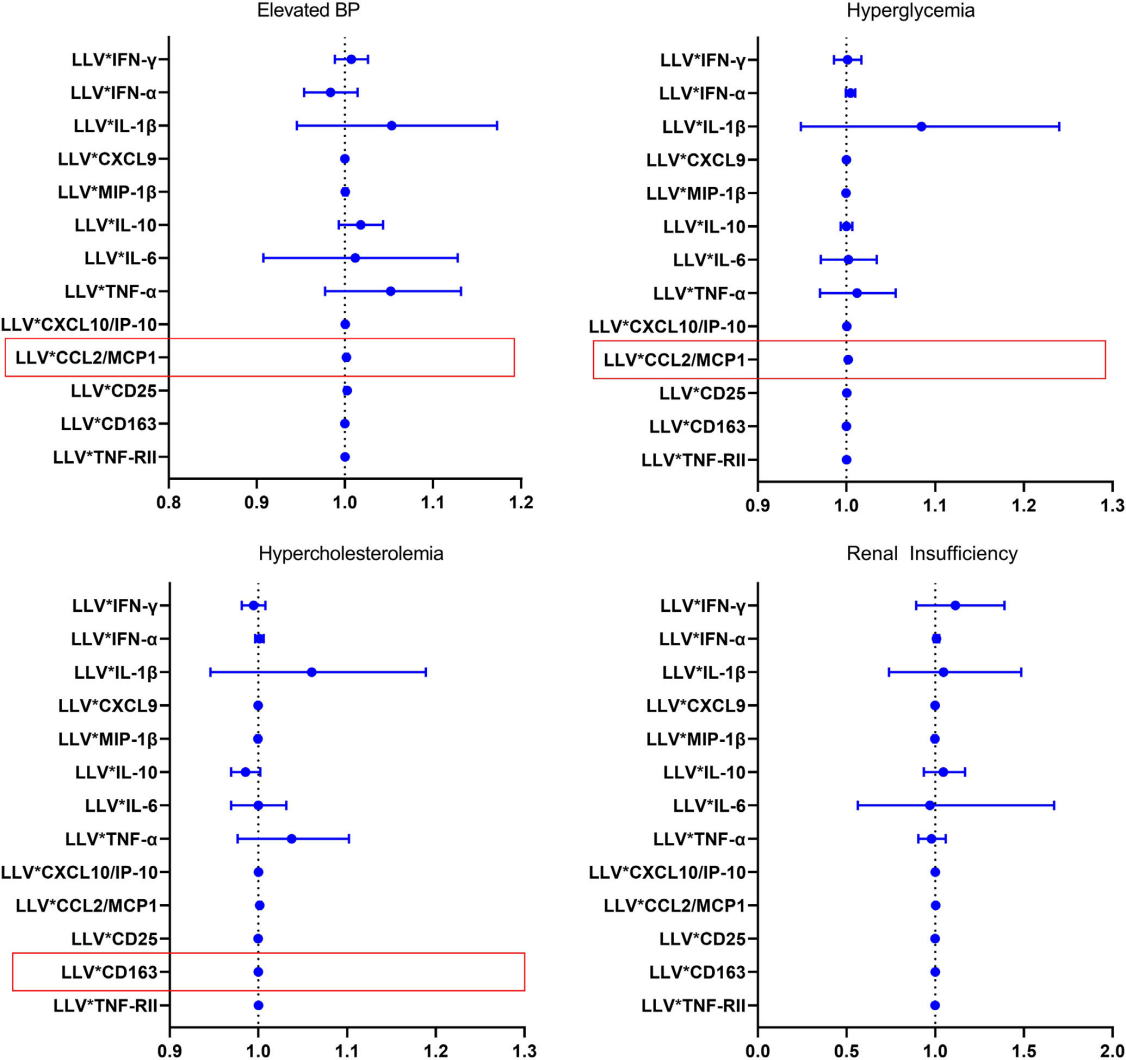
Interaction between low‐level viraemia and immune activation marker in the association with elevated blood pressure, hyperglycaemia, hypercholesterolemia and renal insufficiency at the enrolment visit. Interaction between low‐level viraemia (LLV) and each immune activation marker in the association with select non‐infectious comorbidity. Each row is a separate model.

In unadjusted analyses, there was a statistically significant interaction between LLV and TNF‐α, CCL2/MCP‐1 and TNF‐RII in the association with any NCD at the enrolment visit (Figure 3). Examining each NCD individually, there was a significant interaction between LLV and CCL2/MCP‐1 in the association with elevated BP and hyperglycaemia. The LLV and CCL2/MCP‐1 interaction was borderline significant, while the interaction between LLV and CD163 was statistically significant in the association with hypercholesterolemia (Figure 4).

## 4 DISCUSSION

In this longitudinal study of ART‐experienced PLWH, we found that pLLV was associated with an increased risk of developing NCDs. The relationship was particularly pronounced when restricting to participants on dolutegravir, which is now the standard of care for these PEPFAR clinics. These findings highlight the need to intensify case management of viral loads in the range of 50−999 c/ml which is already being done in some of these countries such as Tanzania.

While we found a significant association with pLLV and any NCD, the association was not significant for any of the NCDs examined separately. Potentially, this is due to limited power with the adjustment for several confounders and the low number of participants with pLLV and NCDs. Other studies have also found that LLV was not associated with NCDs. In a Swedish cohort, there was a significant association with a viral load >1000 c/ml but not a significant association with LLV and cardiovascular disease [19]. Another study among PLWH in South Africa similarly found that having LLV did not increase the odds of cardiovascular risk [20]. Our study is among the first to examine the association with LLV at multiple time points and assess other NCDs such as elevated BP and hyperglycaemia, although others have found an association with cumulative viral load and hypertension [21].

Interestingly, when restricting to participants on dolutegravir, we found significant associations between pLLV and hypercholesteremia and renal insufficiency, while the association with hyperglycaemia was borderline significant. Non‐nucleotide reverse transcriptase inhibitors such as efavirenz and nevirapine are associated with an increased risk of dysglycaemia and dyslipidaemia [22], while PIs are associated with dyslipidaemia, insulin‐resistance, cardiovascular disease and cerebrovascular disease [23]. In contrast, dolutegravir is associated with an increased risk of hypertension [24]. Dolutegravir is also more efficacious in achieving viral suppression compared to older regimens and thus we may see lower rates of pLLV [25]. Therefore, it is possible there is residual confounding, and that the ART regimen effect may be diluting the association between pLLV and NCDs.

In our secondary analysis restricting to the subset of participants with immune activation data at the enrolment visit, we identified an interaction between LLV and the TNF pathway as well as CCL2/MCP‐1. Other studies have illustrated that biomarkers of immune activation remain elevated during pLLV and may be a pathway to NCDs [26]. TNF associations with the development of cardiovascular disease and viral replication are reported in the literature [16, 27]. We also saw significant interactions of LLV and MCP‐1 in the association with any NCD, elevated BP and hyperglycaemia. MCP‐1 has been associated with worse HIV outcomes and neurocognitive functioning [28, 29]. Supporting our findings, prior work among a French cohort of PLWH aged ≥75 years also found that MCP‐1 was associated with developing a comorbidity [27].

Strengths of these analyses include the multicounty setting and 10 years of follow‐up for some participants. However, these analyses are not without some limitations. We were unable to account for diet or exercise that play a role in the development of many of the NCDs and could be an important confounder in the association between pLLV and NCDs. Additionally, for the primary sensitivity analysis and secondary analysis, we utilized slightly different populations. In the secondary analysis examining biomarkers and LLV interaction, we only had access to a subset of cross‐sectional enrolment data prior to 2018. Dolutegravir was systematically rolled out to PEPFAR clinics beginning in late 2018 and, therefore, the main analyses used data from 2013, while the sensitivity analysis restricting to those on dolutegravir only examined participants with visits since late 2018. This limitation also meant we were unable to measure the interaction of LLV with biomarkers among participants on dolutegravir which differed in our primary analysis.

## 5 CONCLUSIONS

These findings suggest a potential mechanism for the development of NCDs among individuals with LLV. Aggressive management of LLV may positively impact NCDs among PLHIV. Targeting viral suppression below the limit of detection on clinical HIV viral load assays may reduce the risk of non‐infectious complications of HIV.

## COMPETING INTERESTS

The authors declare no competing interests.

## AUTHORS’ CONTRIBUTIONS

ALE and JAA conceived of the presented research idea. ALE designed the statistical model, analysed the data and authored the first draft of the research manuscript. NJ, ND and SC provided guidance and input on the statistical model. EB, VS, HK, JM, HS and MI carried out the data collection, laboratory activities and reviewed the collected data for quality and reliability. AE, BS, TAC, ND, NJ and SC contributed to the interpretation of the results. JAA, NS and CSP provided overall direction and planning for the AFRICOS study. All authors provided critical feedback and helped shape the research, analysis and manuscript. The author(s) read and approved the final manuscript.

## FUNDING

This work was supported by the President's Emergency Plan for AIDS Relief via a cooperative agreement between the Henry M. Jackson Foundation for the Advancement of Military Medicine, Inc. and the U.S. Department of Defense [W81XWH‐11‐2‐0174, W81XWH‐18‐2‐0040].

## DISCLAIMER

The views expressed are those of the authors and should not be construed to represent the positions of the U.S. Army or the Department of Defense. The investigators have adhered to the policies for the protection of human subjects as prescribed in AR 70–25.

## Data Availability

The data sets generated and/or analysed during the current study are not publicly available due to privacy protections but are available from the corresponding author on reasonable request. The Henry M. Jackson Foundation for the Advancement of Military Medicine (HJF) and the Water Reed Army Institute of Research (WRAIR) are committed to safeguarding the privacy of research participants. Distribution of data will require compliance with all applicable regulatory and ethical processes, including the establishment and approval of an appropriate data‐sharing agreement. To request a minimal data set, please contact the data coordinating and analysis centre (DCAC) at PubRequest@hivresearch.org and indicate the RV329 study along with the name of the manuscript.
